# Specialized metabolism gets a MED regulator

**DOI:** 10.1093/plphys/kiag099

**Published:** 2026-02-26

**Authors:** Praveen Khatri

**Affiliations:** Assistant Features Editor, Plant Physiology, American Society of Plant Biologists; Department of Biology, University of Toronto at Mississauga, Mississauga, ON, Canada L5L 1C6

Plants are sessile organisms. They have developed a plethora of physiological strategies to cope with biotic and abiotic stresses. Among these physiological adaptations, they have evolved the ability to produce specific organic compounds referred to as specialized metabolites. These metabolites are biosynthesized from simple precursors derived from primary metabolism and play critical roles in plant defense, communication, and environmental adaptation. The major classes of specialized metabolites include terpenoids, alkaloids, glucosinolates, cyanogenic glycosides, and phenolic metabolites such as flavonoids (Huang and Dudareva 2023). Apart from their vital role in plant physiology, these specialized metabolites have attracted considerable attention during the past several decades due to their broad applications in human and livestock health and the increasing importance for sustainable agriculture practices (Singh et al. 2023; Khatri et al. 2025). Thus, understanding how plants regulate the biosynthesis of specialized metabolites is of broad biological, ecological, and practical significance.

Jasmonic acid (JA) is a central phytohormone in plant innate immunity and stress responses and plays a key role in activating specialized metabolic pathways. JA signaling is well known to induce the production of terpene indole alkaloids (TIAs) and flavonoids by relieving transcriptional repression and enabling pathway-specific transcription factors to activate biosynthetic genes. Numerous regulators of JA-responsive specialized metabolism are known, including MYC2 in alkaloid biosynthesis and GL3, MYB12, and MYB111 in flavonoid regulation (Ma and Constabel 2019; Hou et al. 2023). While studies of these regulators have greatly advanced our understanding of pathway-specific control, far less is known about how general transcriptional machinery integrates hormonal signaling with specialized metabolic gene expression.

The Mediator complex is a conserved multi-subunit regulatory assembly that serves as a molecular link between transcriptional activators and RNA polymerase II. MED25, a subunit of the Mediator tail module, has been extensively studied for its roles in plant growth and development, including flowering time, cell proliferation, and responses to multiple phytohormones and environmental stresses. In particular, MED25 has been implicated in JA signaling pathways (Çevik et al. 2012). However, whether MED25 directly participates in the regulation of specialized metabolic gene programs downstream of JA signaling had remained unclear.

In this issue of *Plant Physiology*, Singleton et al. 2026 address this question by examining the role of MED25 in 2 evolutionarily and chemically distinct specialized metabolic pathways: TIA biosynthesis in the medicinal plant *Catharanthus roseus* and flavonoid biosynthesis in the model plant *Arabidopsis thaliana*. The authors show that perturbation of MED25 reshapes pathway regulation and metabolic output in both *C. roseus* and *Arabidopsis*, using a combination of gene silencing, transcriptomic analyses, and metabolite profiling in *C. roseus* hairy roots and loss-of-function mutants in *Arabidopsis*. In parallel, protein–protein interaction assays were employed to dissect how transcriptional regulators associate with MED25 and to identify the functional domains responsible for these interactions.

The study demonstrates that MED25 functions as a transcriptional co-activator of specialized metabolism. In *C. roseus*, MED25 was found to be ubiquitously expressed and localized to the nucleus but was not transcriptionally induced by JA, consistent with a role as a constitutive co-regulator rather than a hormone-responsive gene. Silencing of *CrMED25* resulted in significant changes in the regulation of the TIA pathway, including altered expression of key transcriptional activators such as *CrMYC2* and *ORCA6*. At the metabolic level, silencing *CrMED25* decreased the levels of the major TIAs ajmalicine (Corynanthe-type) and catharanthine (Iboga-type), while levels of tabersonine (Aspidosperma-type) remained unaffected. This metabolic complexity indicates that *MED25* does not globally downregulate the entire pathway but rather modulates specific branches of the TIA biosynthetic pathway. Mechanistically, the authors show that MED25 directly interacts with CrMYC2, a key regulator of JA-induced expression of genes involved in TIA biosynthesis. The study also revealed that the conserved activator interaction domain (ACID) of MED25 is sufficient for interaction with CrMYC2, which adds to our understanding of structural basis of MED25 interaction with other regulators.

The authors also reported a similar regulatory role of MED25 in *Arabidopsis*. They found that the loss of MED25 function impaired the JA-mediated activation of flavonoid and downstream anthocyanin biosynthesis pathway. In the *Arabidopsis* mutant line (*med25*) the JA-induced anthocyanin accumulation was significantly reduced, along with the decreased expression of both early and late flavonoid biosynthesic pathway genes. The authors further show that MED25 interacts with GLABRA3 (GL3), a key bHLH transcription factor in flavonoid biosynthesis, as well as with the MYB transcription factors MYB12 and MYB111, which regulate different stages of the pathway. Consistent with their findings in *Catharanthus*, the authors demonstrate that the ACID domain of *Arabidopsis* MED25 is sufficient for interaction with GL3, suggesting the evolutionary conservation of the function of MED25 as a co-regulatory platform between transcription factors and the core transcriptional machinery.

Collectively, these results demonstrate that MED25 is a conserved transcriptional hub that integrates jasmonate signaling with specialized metabolic processes in different pathways and species. In contrast to a pathway-specific transcriptional regulator, MED25 is a general component of the transcriptional machinery that affects metabolic output in response to hormonal signals. In this regard, MED25 is the latest addition to a growing list of Mediator subunits, including MED5, which was shown to play a role in phenylpropanoid metabolism (Bonawitz et al. 2012), suggesting a more general role for Mediator in the regulation of plant metabolic diversity. The study by Singleton et al. (2026) greatly advances our understanding of the role of global transcriptional regulators in the regulation of specialized metabolism and suggests MED25 as a leverage point for future metabolic engineering.

The next steps in exploring the role of MED25 include determining whether MED25 interacts with other transcription factors in a simultaneous or sequential manner. Such interaction studies would answer the crucial dynamics of Mediator recruitment. It is also an open question how MED25 controls early and late events in flavonoid biosynthesis. Finally, it is unknown if this mode of MED25 recruitment is also present in other specialized metabolic pathways. Interestingly, other Mediator subunits have already been implicated in various physiological processes. For example, MED2, MED5, and MED23 are known for their role in phenylpropanoid pathway, and MED16 is known to affect defense gene expression in both salicylic acid– and jasmonate-responsive pathways (Wathugala et al. 2012; Dolan et al. 2017). Placed in this broader Mediator landscape, MED25 provides a framework for understanding how plants integrate hormone responses with transcriptional programs that generate and constrain metabolic diversity.

## Data Availability

No new data were generated or analyzed for this article.
